# Coronary computed tomographic angiography derived findings and risk score improves the allocation of lipid lowering therapy compared to clinical score

**DOI:** 10.1097/MD.0000000000028801

**Published:** 2022-02-11

**Authors:** Biyanka Jaltotage, Ashu Gupta, Umar Ali, Gavin Huangfu, Jamie Rankin, Richard Parsons, Girish Dwivedi

**Affiliations:** a Department of Cardiology, Fiona Stanley Hospital, Perth, Western Australia, Australia; b Department of Radiology, Fiona Stanley Hospital, Perth, Western Australia, Australia; c Department of Cardiothoracic Surgery, Fiona Stanley Hospital, Perth, Western Australia, Australia; d School of Pharmacy, Curtin University, Perth, Western Australia, Australia; e Harry Perkins Institute of Medical Research, University of Western Australia.

**Keywords:** CCTA, cohort risk score, risk prediction, risk reclassification, statin allocation

## Abstract

The initiation of therapy for atherosclerotic cardiovascular disease (ASVCD) is currently guided by cohort-based risk scores. Coronary computed tomographic angiography (CCTA) offers more personalised risk assessments to optimise therapy allocation. This study investigates the utility of CCTA determined coronary stenosis (both obstructive and non-obstructive plaque) to guide allocation of lipid lowering therapy. A retrospective analysis of 450 patients with CCTA performed for the assessment of chest pain at a single centre was conducted. Baseline characteristics, investigations, treatments and clinical outcomes were recorded. The allocation of lipid lowering therapy was evaluated with three models, cohort-based risk score (pooled cohort equation), a previously validated CCTA based clinical risk score (pooled cohort equation and CCTA findings) and CCTA alone (without clinical characteristics). The reclassification analysis included 266 patients. Compared to the cohort-based risk score, CCTA based clinical risk score in total reassigned 23% of patients. CCTA alone compared to the CCTA based clinical risk score correctly reassigned 23% and incorrectly reassigned 10%. When comparing the performance of CCTA alone against the cohort-based risk score, both the additive NRI of 25.8 (95% CI 4.12–37.56) and absolute NRI of 13.2 (95% CI 5.88–19.77) was significant. Revascularisation was required in 3% with a low cohort-based risk, but no patients with low risk as per CCTA alone or CCTA based clinical risk score required revascularisation The use of a CCTA based clinical risk score or CCTA alone compared to cohort-based risk scores can improve the allocation of lipid lowering therapy.

## 1 Introduction

More than 28% of all individuals over the age of 40 in the United States are using lipid lowering therapies for the treatment of atherosclerotic cardiovascular disease (ASCVD).^[1]^ With globally increasing rates of metabolic syndrome the prescription of these medications will continue to increase.^[2]^ The initiation of lipid therapy for primary prevention is traditionally guided by the use of cardiovascular risk calculators such as the pooled cohort equation (PCE) developed by the American College of Cardiology and American Heart Association. This equation is a cohort-based calculator used to estimate the 10-year risk of ASCVD.^[3]^ More recently coronary computed tomographic angiography (CCTA) has emerged providing a rapid non-invasive assessment of coronary stenosis and coronary artery calcification (CAC), a measure of overall plaque burden. As CCTA becomes more widely utilised, an opportunity exists to offer patients a more personalised assessment of ASCVD risk to optimise allocation of lipid lowering therapy. Previous studies investigated the use of CAC and non-obstructive plaque to determine appropriate allocation, with reclassification in up to 14% of patients when compared to traditional cohort-based risk scores.^[4–6]^ However, these studies may have underestimated the real-life value of CCTA in lipid therapy allocation as patients with obstructive disease were excluded. Due to the high utilisation rates of these therapies even incremental improvements in the allocation of therapy could change management in a great number of patients.

A composite of CCTA and cohort-based risk score to form a CCTA based clinical risk score, despite the added complexity, holds the most promise in improving the allocation of lipid lowering therapy. A model based on CCTA alone to determine allocation of therapy increases simplicity but has never been investigated. This study investigates the utility of CCTA determined coronary stenosis (obstructive and non-obstructive plaque) in conjunction with cohort-based risk score or alone to guide allocation of lipid lowering therapy.

## 2 Materials and methods

### 2.1 Study population

A retrospective analysis was conducted of a local patient database with CCTA performed for the assessment of chest pain between 2015 and 2018 at a tertiary hospital. Both inpatients and outpatients were included in this database, and patients were excluded if their primary care was in private practice. Local ethics approval was provided, and the study was registered as a Quality Improvement Activity (No. 24929).

### 2.2 Study design

The 10-year ASCVD risk derived by the PCE was calculated for each patient. Following ACC and AHA guidelines patients with 10-year ASCVD risk >7.5% were deemed to be eligible for lipid lowering therapy.^[7]^ If clinical details or results needed to calculate 10-year cardiovascular risk were unavailable, these patients were excluded from the reclassification data. However, they were not excluded from the baseline characteristic and revascularisation data. Based on CCTA derived coronary stenosis patients were placed into one of four categories, normal—pristine coronary arteries without stenosis, mild—<50% stenosis, moderate—50% to 70% stenosis and severe—over 70% stenosis. All patients with obstructive plaque, defined as >50% stenosis (moderate and severe group), were deemed to be appropriate for lipid lowering therapy, regardless of their calculated 10-year ASCVD risk. For patients with non-obstructive plaque (<50% stenosis [mild group], and no plaque [normal group]) we employed the reclassification thresholds described by Emami et al to determine lipid therapy eligibility.^[5]^ These thresholds to modify ASCVD risk were formulated following a meta-analysis of published data on the prognostic value of non-obstructive plaque. In brief, females with an ASCVD risk score between 4.4% and 7.4% and males with risk scores between 4.6% and 7.4%, with non-obstructive plaque on CCTA had risk scores revised to over 7.5%. Females with risk scores between 7.5% and 13.7% and males with risk scores between 7.5% and 14.3%, but normal CCTA without plaque had their risk scores revised to <7.5%.^[5]^ Patients in the normal group with an original ASCVD risk score over 7.5% but subsequently had a revised risk score <7.5% were labelled as reassigned down. On the other hand, patients in the mild group with an original risk score <7.5% but with a revised score of >7.5%, and those in the moderate and severe groups with an original risk score <7.5%, were labelled as reassigned up.

### 2.3 Clinical outcomes

Outcome data including revascularisation, readmission for acute coronary syndrome (ACS) and death within 12 months were all recorded. Post CCTA revascularisation included either percutaneous coronary intervention or coronary artery bypass grafting.

### 2.4 Data collection and statistical analysis

Patient medical records were reviewed, data collected included patient demographics, cardiovascular risk factor history, type of chest pain at presentation, blood pressure, troponin, lipid panel, CCTA results and follow up coronary artery assessments (stress electrocardiogram [ECG], stress echocardiography, nuclear myocardial perfusion imaging and coronary imaging). The type of chest pain was recorded to be typical, atypical or non-specific following the Diamond-Forrester approach.^[8]^ ECG changes were defined as the presence of T wave inversions or ST depression documented in the medical record. Troponin was positive if the result was above the assay reference range. All data was recorded with blinding to outcome data.

We also investigated the efficacy of CCTA alone to appropriately allocate lipid lowering therapy. To assess performance, the net reclassification index (NRI) was calculated by comparing classification by CCTA findings alone (absence of plaque, non-obstructive plaque or obstructive plaque) and PCE clinical risk against the CCTA based clinical risk score.^[9]^

## 3 Results

**Figure 1:**
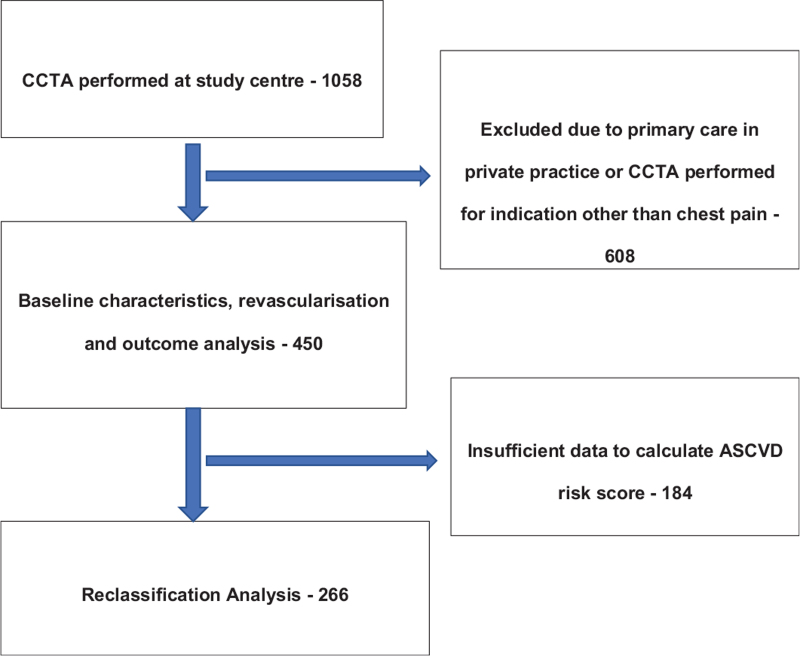
Study design and final patient populations. CCTA denotes coronary computed tomographic angiography and ASCVD atherosclerotic cardiovascular disease.

In total 1058 patients had CCTA performed at our study center between 2015 and 2018. Six hundred eight patients were excluded as their ongoing care was in private practice or CCTA was performed for an indication other than the assessment of chest pain. A total of 450 patients remained and were included in the study for baseline characteristic, revascularisation and outcome analysis. A further 184 patients were excluded due to insufficient data to calculate the 10-year ASCVD risk score. In total, 266 patients were included for reclassification analysis (Fig. 1).

Baseline characteristics are described in Table 1. Half (48.9%) of patients had a normal CCTA, a quarter (24.2%) had mild stenosis, and a few had moderate (9.4%) or severe (17.5%) stenosis. There was a significant increase in age, proportion of males and presence of dyslipidaemia with increasing stenosis severity. Patients with severe stenosis on CCTA were less likely to present with non-specific chest pain. The CAC and calculated ASCVD risk score were associated with increased stenosis severity. Of note, a CAC of zero was recorded in 186 (83.4%), 19 (17.6%), 7 (16.3%), and 11 (14.5%) patients in the normal, mild, moderate and severe groups respectively.

**Table 1:** Baseline characteristics.

	Normal (n = 223)	Mild (n = 108)	Moderate (n = 43)	Severe (n = 76)	Normal vs severe
Demographics					
Age (years)	47.9	57.3	60	59.5	***P* < .01**
Male	36.3%	41.7%	48.8%	54%	***P* < .01**
Presentation					
Non-specific chest pain	66.4%	58.3%	55.8%	36.8%	***P* = .03**
Atypical chest pain	29.2%	30.6%	27.9%	46%	*P* = .20
Typical chest pain	4.48%	11.1%	16.3%	17.1%	*P* = .34
Risk Factors					
Hypertension	30%	50.9%	48.8%	55.3%	*P* < .06
Dyslipidaemia	33.6%	55.6%	60.5%	67.1%	***P* = .01**
Smoking history	41.7%	44.4%	60.5%	50%	*P* = .53
Diabetes	8.1%	20.4%	25.6%	13%	*P* = .71
Family history	36.8%	28.7%	37.2%	35.5%	*P* = .92
Investigations					
Troponin	13.9%	14.8%	4.7%	15.8%	*P* = .69
ECG changes	26.9%	34.3%	11.6%	35.5%	*P* = .15
Personalised Risk					
ASCVD 10-year risk score	4.8%	12.9%	13.8%	13.5%	***P* < .01**
Calcium score	1.2	54.2	179.2	255.6	***P* < .01**

ECG denotes electrocardiography and ASCVD atherosclerotic cardiovascular disease. Bolding highlights statistical significance with a p value of <0.05

Of the 450 patients in this study, 115 patients had subsequent testing following CCTA with 11 patients having more than one test. Coronary angiography (22%) was performed most frequently, followed by nuclear myocardial perfusion imaging (4%), stress echocardiography (1.3%) and stress ECG (0.7%). As seen in Table 2 most testing was performed in those with more severe stenosis. In total, 28 (6.2%) patients were revascularized, eight of these patients had a 10-year ASCVD risk score <7.5% and two had a calcium score of zero. ACS (1.1%) was rare and almost all patients were alive at 1 year (98.2%).

**Table 2:** Further investigations following coronary computed tomographic angiography and outcomes.

	Normal (n = 223)	Mild (n = 108)	Moderate (n = 43)	Severe (n = 76)
Investigations				
Stress ECG	0%	0.9%	1.9%	4.6%
Stress echocardiography	0%	1.9%	7.0%	1.3%
Nuclear MPI	0%	4.6%	11.6%	10.5%
Coronary angiogram	0%	12.4%	48.8%	85.5%
Outcomes				
PCI/CABG	0%	0%	7%	32.9%
ACS	0%	2.8%	2.3%	1.3%
Alive at 1 year	99.1%	96.3%	97.7%	98.7%

ACS = acute coronary syndrome, CABG = coronary-artery bypass grafting, ECG = denotes electrocardiography, MPI = myocardial perfusion imaging, PCI = percutaneous coronary intervention.

**Figure 2:**
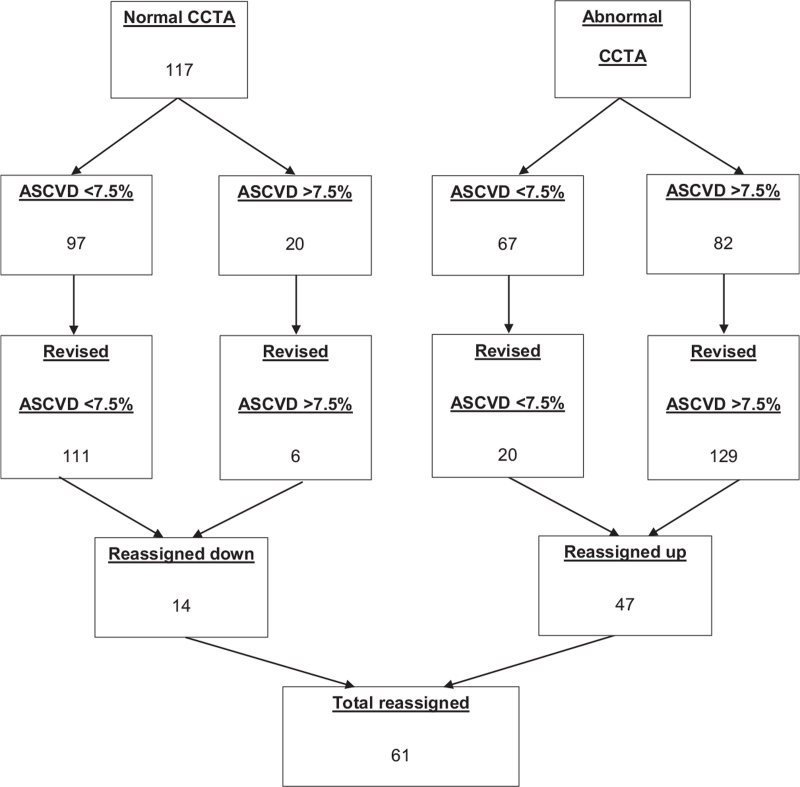
Reclassification of the original pooled cohort equation risk by the revised risk score. CCTA denotes coronary computed tomographic angiography and ASCVD atherosclerotic cardiovascular disease.

ASCVD risk was calculated in 266 patients, of which 102 had a 10-year risk >7.5%. However, when the CCTA based clinical risk score was calculated 135 patents had a risk score >7.5%. As seen in Figure 2 a total of 61 (22.9%) patients were reassigned, 14 (5.3%) were reassigned down and 47 (17.7%) reassigned up. The ability of CCTA alone to reclassify the original PCE derived clinical risk score in comparison to the CCTA based cohort risk score by Emami et al is displayed in Table 3. CCTA alone reassigned 87 (32.7%) patients in total, 61 (22.9%) were correctly reassigned, and 26 (9.8%) were incorrectly reassigned in comparison to the CCTA based clinical risk score.

**Table 3:** Risk reclassification of pooled cohort equation calculated risk by coronary computed tomographic angiography in comparison to the revised risk score.

	Revised risk > 7.5%	Revised risk < 7.5%		
Correctly reassigned	47	14	**Additive NRI** 25.8	95% CI (4.11–37.56)
Incorrectly reassigned	6	20	**Absolute NRI** 13.2	95% CI (5.88–19.77)
Net reclassification	41	−6		

NRI denotes net reclassification index.

To evaluate the performance of CCTA alone against the PCE clinical risk score to allocate lipid lowering therapy a novel statistical tool was employed, the NRI. For background the additive NRI ranges from 200 (all patients with events had higher risk prediction and all patients without events had lower risk prediction with the new model) to −200 (opposite is true) and does not reflect a proportion. The absolute NRI ranges from −100% to 100% and represents the proportion of correctly and incorrectly reclassified patients.^[9]^ Both the additive NRI of 25.8 (95% CI 4.12–37.56) and absolute NRI of 13.2 (95% CI 5.88–19.77) were significant, suggesting that a CCTA alone model improved reclassification compared to the PCE clinical risk score.

## 4 Discussion

Cardiovascular risk calculators, such as the PCE, currently form the foundation of ASCVD risk assessment for primary prevention.^[3]^ These risk calculators are effective tools at describing cohort risk. However, with the increasing availability of CCTA we now have the opportunity to provide patients with a more personalised risk to guide treatment. This study investigates the role of CCTA in revising calculated ASCVD risk to improve the allocation of lipid lowering therapy.

The findings highlight established risk factors such as age, male gender and dyslipidaemia as significant predictors of coronary stenosis severity, on the other hand presentation with non-specific chest pain was protective. Interestingly, other traditional risk factors including hypertension, smoking, diabetes and a family history of ischaemic heart disease were not found to be significant in this cohort. This may be a consequence of CCTA utilisation in low to intermediate risk groups, while higher risk groups with multiple predisposing factors proceeded directly to more definitive coronary angiography. This may also explain why abnormal troponin levels and ECG changes were not predictive of stenosis severity. Individuals with elevated troponin levels but non-obstructive plaque may represent myocardial infarction with non-obstructive coronary arteries, however troponin rises due to other causes such as heart failure may also be possible and cannot be differentiated from this data. Lastly, several patients also presented with typical chest pain but still underwent CCTA, again likely representing a group with limited risk factors and a lack of ECG changes, this is somewhat confirmed by typical chest pain not being predictive of stenosis severity. A subset of individuals with typical chest pain but non-obstructive plaque (n = 22) could be reclassified as having angina with no obstructive coronary artery disease.

Following CCTA if further investigation was required, coronary angiography was the most frequently employed test. Stress ECG, stress echocardiogram and nuclear myocardial perfusion imaging in comparison were rarely utilised. Surprisingly high rates of secondary investigations were observed in the mild group, particularly coronary angiography. This was largely a result of non-diagnostic CCTA imaging that was unable to exclude severe stenosis, none of these patients required revascularization. Predictably, the highest rates of revascularization were in the severe group. The rates of ACS and death were similar to previous studies, confirming the low to intermediate risk nature of the study population.^[10]^

A CAC of 0 was present in 14.5% of patients in the severe group and two patients requiring revascularisation. These findings raise concerns regarding a reliance on CAC and the power of zero to guide preventative treatment decisions.^[4,11]^ In addition, 24 patients in the severe group had an original risk score of <7.5%, 8 of whom required revascularisation, demonstrating the limitations of cohort-based risk scores. As seen in Figure 2 the CCTA based clinical risk score was effective in reassigning patients, in total 61 patients (22.9%) were reassigned (47 reassigned up and 14 reassigned down). No patient with a CCTA based clinical risk score of <7.5% required revascularisation. These results do suggest that CCTA based reclassification may improve treatment of ASCVD, and that previous work may have underestimated its benefit.^[5]^ Furthermore, given the added complexity of a CCTA based clinical risk score, the ability of CCTA alone to accurately distribute lipid lowering therapy was investigated. The NRI assessed the performance of the CCTA alone model against the PCE risk score to allocate treatment compared to the CCTA based clinical risk score. The results in Table 3 demonstrate a significant additive and absolute NRI, suggesting an approach with CCTA alone provides incremental value in the allocation of lipid therapy above what risk scores such as the PCE can offer. Overall, these findings support the incorporation of CCTA as part of clinical decision making for ASCVD.

A number of limitations with this study do need to be acknowledged. This data is retrospective and from a single site, and as such has inherent limitations. The study population included symptomatic patients presenting with chest pain to a tertiary hospital and may not represent a truly asymptomatic primary prevention population. It should also be considered that patients with obstructive disease on CCTA are no longer receiving primary prevention treatment due to their burden of ASCVD. Other limitations include the reduction in reclassification analysis population in comparison to the original study population, which runs the risk of introducing a selection bias. In addition, due to limited data we were unable to consider the impact of high-risk plaques which have emerged as robust predictors of ASCVD risk.^[12]^ Further prospective studies are still required investigating the use of CCTA guided lipid allocation to demonstrate improved outcomes.

## 5 Conclusion

The findings demonstrate the use of CCTA either as part of a clinical risk score or alone, may improve the allocation of lipid lowering therapy beyond what a cohort based clinical risk score can offer, and could reduce the likelihood of classifying patients with severe ASCVD as low risk.

## Author contributions

**Conceptualization:** Ashu Gupta, Girish Dwivedi.

**Data curation:** Umar Ali, Gavin Huangfu, Girish Dwivedi.

**Formal analysis:** Biyanka Jaltotage, Gavin Huangfu.

**Investigation:** Biyanka Jaltotage, Umar Ali, Gavin Huangfu, Girish Dwivedi.

**Project administration:** Jamie Rankin, Girish Dwivedi.

**Resources:** Jamie Rankin, Richard Parsons, Girish Dwivedi.

**Supervision:** Ashu Gupta, Jamie Rankin, Richard Parsons, Girish Dwivedi.

**Visualization:** Girish Dwivedi.

**Writing – original draft:** Biyanka Jaltotage.

**Writing – review & editing:** Biyanka Jaltotage, Ashu Gupta, Richard Parsons, Girish Dwivedi.
